# Antioxidant, Anti‐Inflammatory, and Immunomodulatory Benefits of DDGS in Broiler Growth and Meat Quality

**DOI:** 10.1155/vmi/2796378

**Published:** 2026-09-06

**Authors:** Ayman S. Salah, Ayman E. Taha, Rasha I. M. Hassan, Esraa M. Mohamed, Asmaa H. Abd Elkareem, Afnan H. Saaty, Mahmoud Alagawany, Khan Md. Shaiful Islam, Hala Y. Amer

**Affiliations:** ^1^ Department of Nutrition and Clinical Nutrition, Faculty of Veterinary Medicine, New Valley University, P. O. Box: 72511, El-Kharga, Egypt, nv.aun.edu.eg; ^2^ Department of Public Health, College of Veterinary Medicine, King Faisal University, Al-Ahsa 31982, Saudi Arabia, kfu.edu.sa; ^3^ Department of Nutrition and Clinical Nutrition, Faculty of Veterinary Medicine, Assiut University, Assiut, Egypt, aun.edu.eg; ^4^ Department of Food Hygiene, Safety and Technology, Faculty of Veterinary Medicine, New Valley University, El Kharga, Egypt, nv.aun.edu.eg; ^5^ Department of Physiology, Faculty of Veterinary Medicine, New Valley University, P. O. Box: 72511, El Kharga, Egypt, nv.aun.edu.eg; ^6^ Food and Nutrition Department, Human Sciences and Design, King Abdulaziz University, Jeddah 21589, Saudi Arabia, kau.edu.sa; ^7^ Poultry Department, Faculty of Agriculture, Zagazig University, Zagazig 44511, Egypt, zu.edu.eg; ^8^ Department of Animal Nutrition, Bangladesh Agricultural University, Mymensingh 2202, Bangladesh, bau.edu.bd

**Keywords:** blood metabolites, DDGS, economic efficiency, meat quality, performance

## Abstract

This study aimed to evaluate the effects of corn‐based distiller’s dried grains with solubles (DDGS) on various parameters in broilers, including immune modulation, antioxidant and anti‐inflammatory effects, growth performance, carcass traits, blood hematology, serum parameters, meat quality, and economic efficiency. A total of 150 one‐day‐old broiler chicks were assigned to five groups, including a control and four groups with increasing levels of DDGS (8%, 16%, 24%, and 32%). The inclusion of DDGS significantly improved growth performance (*p* < 0.05) without affecting carcass traits (*p* < 0.05). DDGS supplementation led to a significant increase in the weight of immunological organs, particularly the bursa of Fabricius (*p* < 0.05), and altered hematological parameters (*p* < 0.05), including RBCs, WBCs, Hb, and PCV. Serum total protein and globulin levels were increased (*p* < 0.05), while uric acid levels decreased (*p* < 0.05). Lipid profiles also improved with reductions in triglycerides, cholesterol, and HDL‐C (*p* = 0.001), and thyroid hormones G3 and G4 increased (*p* < 0.001). DDGS supplementation enhanced antioxidant and immune markers, including glutathione peroxidase (GPX), superoxide dismutase (SOD), IL‐10, and IgA, IgG, and IgM, while decreasing malondialdehyde (MDA) and interferon‐gamma (IFN‐γ) (*p* < 0.05). Meat quality was slightly improved in terms of dry matter and ash content, without changes in protein or fat levels. In addition, DDGS inclusion reduced feed costs, enhancing economic efficiency. In conclusion, DDGS can be included up to 32% in broiler diets as a cost‐effective protein source, improving health parameters and meat quality without adverse effects.

## 1. Introduction

The expansion of poultry production plays a vital role in mitigating the growing nutritional demands by supplying billions of people with essential proteins, vitamins, and minerals across the globe [1]. The development of commercial strains with enhanced feed conversion ratio (FCR) and shorter production cycles has greatly improved poultry productivity, particularly in meat and egg production [2]. However, feed itself remains a significant factor in poultry farming, accounting for approximately 70% of the total production cost [3]. Among the various feed ingredients used in poultry diets, soybean meal is a key source of protein, which is also more expensive than other nutrients [4]. However, price fluctuations and competition between human and animal consumption of soybeans have motivated researchers to explore alternative sources of protein to include in their diet [5].

In response to the rising costs, there is an increasing interest in partially replacing common ingredients with alternatives, such as sunflower meal, distiller’s dried grains with solubles (DDGS), canola meal, guar meal, millets, cottonseed meal, and various nutritional additives. These alternatives include substances such as curcumin [6], grape seeds, *Arthrospira platensis* [7–9], sumac [10], chamomile [11], parsley [12], and probiotics [13], among others. Among these alternatives, DDGS, a byproduct of the ethanol industry, has attracted considerable attention due to its high nutritional value.

DDGS is produced through the dry milling of cereal grains such as corn, barley, wheat, sorghum, and rice. Unlike starch, this method preserves and concentrates the majority of the nutrients from the original grain, producing a product that is rich in protein (20%–30%), fat (8%–12%), vitamins, minerals, and amino acids [14, 15]. Consequently, DDGS serves as a viable alternative to traditional feed components such as corn and soybean meal [16, 17]. In addition to its nutritional content, DDGS also contains bioactive compounds such as mannan oligosaccharides and β‐glucans, which are components of yeast cell walls known to enhance intestinal health and regulate immune responses in poultry through improving gut health by preventing the adhesion and colonization of pathogenic bacteria, promoting the growth of beneficial microbiota, enhancing intestinal integrity, and activating macrophages and other immune cells via pattern recognition receptors, thereby stimulating both innate and adaptive immune responses [17–19].

Previous studies have demonstrated that DDGS, with its elevated levels of protein, energy, vitamins, and essential fatty acids such as linoleic acid and xanthophyll, is a suitable ingredient for poultry feed [3, 20]. Research has also shown that up to 20% of broiler diets can consist of DDGS without negatively affecting the birds’ health, growth performance, or meat quality [3, 21].

Effective strategies for incorporating DDGS into the diets can improve both poultry production and economic feasibility, despite the high costs associated with traditional feed ingredients. However, further research is needed to better understand the physiological impacts of DDGS on broiler health and performance, as well as to determine the optimal inclusion levels for maximum benefit. So, the study was conducted to investigate the effects of corn DDGS on broiler growth, carcass traits, blood hematology, blood biochemical parameters, immunity, antioxidant status, meat quality, and the economic viability of its inclusion in the diet of broilers.

## 2. Materials and Methods

### 2.1. Experimental Design and Management of Birds

The study was conducted using 150 one‐day‐old broiler chicks (Ross 308). After initial body weight determination, the chicks were randomly distributed into five groups, each comprising 30 chicks. Each group was further divided into six replicates, with five chicks per replicate. The treatment groups were as follows: (1) Control group, administered a basal diet; (2) fed DDGS at 8%; (3) fed DDGS at 16%; (4) fed DDGS at 24%; and (5) fed DDGS at 32%. The trial lasted for 35 days, with each group monitored for any health issues daily. The feed ingredients, including corn, soybean, corn gluten, sunflower oil, and DDGS, were purchased from local stores in Egypt and analyzed according to [22] using the AOAC (2011) method, and the chemical composition of DDGS is 35.50% crude protein (CP), 8.90% ether extract (EE), and 16.20% CF. The diets were formulated following NRC (1994) guidelines [23], and the ingredient analysis is presented in Table 1.

**TABLE 1:** Composition and chemical analysis of the experimental diets (starter and finisher diets).

Item	Starter (1–21 days)					Grower–finisher (22–35 days)				
	Groups (% DDGS)					Groups (% DDGS)				
	1 (0)	2 (8)	3 (16)	4 (24)	5 (32)	1 (0)	2 (8)	3 (16)	4 (24)	5 (32)
Ingredient, %										
Yellow corn	53.87	49.20	47.6	45.90	44.37	59.18	55.99	54.28	52.66	50.74
Soybean meal	36	34.50	28.1	21.7	15.00	30.01	26.40	20.00	13.50	7.40
Sunflower oil	2.60	3.70	3.7	3.6	3.50	5.10	5.50	5.50	5.50	5.40
Corn gluten meal	4.00	1.00	0.90	1	1.20	2.15	0.50	0.50	0.50	0.50
DDGS	0.00	8.00	16.00	24.00	32.00	0.00	8.00	16.00	24.00	32.00
Dicalcium P	0.83	0.82	0.82	0.82	0.82	0.82	0.82	0.82	0.82	0.82
Limestone, ground	1.84	1.84	1.85	1.85	1.86	1.64	1.63	1.63	1.64	1.66
Lysine	0.15	0.20	0.30	0.41	0.53	0.32	0.38	0.50	0.61	0.72
Methionine	0.12	0.14	0.14	0.12	0.12	0.18	0.18	0.17	0.17	0.16
Premix^1^	0.30	0.30	0.30	0.30	0.30	0.30	0.30	0.30	0.30	0.30
Common salt	0.30	0.30	0.30	0.30	0.30	0.30	0.30	0.30	0.30	0.30
Total	100	100	100	100	100	100	100	100	100	100
Chemical Composition, %										
Crude protein^2^	23.08	23.12	23.07	23.13	23.15	20.02	20.07	20.09	20.06	20.18
Ether extract^2^	5.01	6.55	7.15	7.64	8.15	7.59	8.51	9.11	9.70	10.20
Crude fiber^2^	3.8	4.84	5.65	6.45	7.24	3.46	4.41	5.21	6.01	6.83
Calcium^3^	1.00	1.00	1.00	1.00	1.00	0.90	0.90	0.90	0.90	0.90
Available P^3^	0.42	0.42	0.43	0.44	0.45	0.41	0.42	0.42	0.43	0.45
Lysine^3^	1.30	1.33	1.31	1.30	1.30	1.30	1.3	1.30	1.30	1.20
Methionine^3^	0.50	0.51	0.51	0.50	0.50	0.50	0.50	0.50	0.50	0.50
ME (kcal/kg diet)^3^	3001	3000	3003	3001	3002	3212	3200	3204	3208	3202

^1^Premix (provided for each kilogram of dietary DM): Vit. A, 6,250,000 IU; Vit.D3, 25,00,000 IU; Vit. E, 25,000 mg; Vit.k3, 1750 mg; Vit.B1, 500 mg; Vit.B2, 2750 mg; Vit.B6, 1250 mg; Vit. B12, 10 mg; nicotinic acid, 20,000 mg; calcium pantothenate, 500 mg; folic acid, 500 mg; biotin, 50 mg; iron, 22 g; copper, 2.5 g; zinc, 37.5 g; manganese, 31 g; iodine, 650 mg; selenium, 113 mg; cobalt, 50 mg).

^2^Analyzed according to AOAC (2011).

^3^Calculated according to NRC (1994).

The trial room was fully disinfected, and a layer of chopped wood shavings was spread on the floor for bedding. To ensure optimal temperature and airflow, each pen was equipped with ventilation fans, water fountains, thermostatically controlled heaters, and cylindrical plastic feeders. All birds were reared under uniform management practices and environmental conditions. Hygienic procedures were strictly followed for the disposal of organic wastes, and free‐choice feed and water were provided to all tested groups throughout the study. The ambient temperature was set at 35°C during the first 3 days of age, and then gradually lowered by 2‐3°C per week till the quails were 5 weeks old. Birds were vaccinated against Newcastle disease at 7 and 18 days using the Hitchner B1 and LaSota strains, and at 14 days, birds were vaccinated against Gumboro disease using the IBD strain. The birds were reared at a relative humidity of 56%–66% and under a 16‐h light/8‐h dark lighting schedule. Mortality was recorded throughout the experimental period.

### 2.2. Performance and Carcass Parameters

The birds were weighed on a weekly basis, and daily records of feed offered and feed refusal were maintained to calculate feed intake (FI). Body weight gain was measured weekly, and FCR was calculated as the feed‐to‐gain ratio weekly [24]. On the final day of the trial, internal organs (heart, gizzard, and liver) and immune organs (thymus, bursa, and spleen) were collected from six randomly selected broilers per replicate. These organs were excised, saline‐rinsed, patted dry, and stripped of any adhered membranes or fat before being weighed separately and expressed as a percentage (%) of the bird’s body weight [25].

### 2.3. Blood Chemistry

At the end of the experimental period (35 days of age), six birds from each treatment group (*n* = 6) were chosen at random, then were anesthetized by using intramuscular injection with 1 mL/kg of ketamine xylazine mixture (2:1), and slaughtered by a sharp knife to complete bleeding, and the blood was collected in a nonheparinized tube. The samples were then pooled into three composite samples (two birds per pool) for subsequent analysis. Serum was isolated by centrifugation at 3000 rpm for 10 min and stored at −18°C until further analysis. The serum samples were tested for total protein and its fractions (albumin and globulin), uric acid, triglycerides, total cholesterol, and high‐density lipoprotein cholesterol (HDL‐C) using an automatic biochemical analyzer (Clima, Ral. Co, Spain), following the protocols outlined in the reagent kit (Pars Azmon Inc., Tehran, Iran). The level of Complement 3 (C3) proteins was determined using an ELISA kit (Lifespan Biosciences, WA, USA). Thyroid hormones, triiodothyronine (T3) and thyroxin (T4), were quantified using an ELISA kit (IBL, Hamburg, Germany) and a photometric detection method. IgA, IgG, and IgM levels were measured using the MAGLUMI 2000 analyzer (Snibe Diagnostics, Shenzhen, China). Interleukin‐10 (IL‐10) and interferon‐gamma (IFN‐γ) were quantified using an ELISA kit (Lifespan Biosciences, WA, USA). The levels of glutathione peroxidase (GPX), malondialdehyde (MDA), and superoxide dismutase (SOD) in serum were measured using a spectrophotometer and commercial test kits (Spectrum, Cairo, Egypt), following the manufacturer’s instructions.

In addition, three more blood samples were collected for hematological analysis and placed in sterile, clearly labeled bottles containing the anticoagulant ethylene diamine tetraacetic acid (EDTA). The samples were analyzed for hematological parameters, including red blood cells (RBCs), white blood cells (WBCs), hemoglobin (Hb), packed cell volume (PCV), heterophils (HETs), lymphocytes (LYMs), monocytes (MONs), eosinophils (ESOs), and basophils (BASs) [26].

### 2.4. Composition of Meat

At 35 days of age, six birds from each treatment group (*n* = 6) were chosen at random, then were anesthetized by using intramuscular injection with 1 mL/kg of ketamine xylazine mixture (2:1), and slaughtered for sample collection. Samples of breast and thigh muscles were collected, vacuum‐sealed, and stored at 4°C for 24 h before analysis. The chemical composition of the meat samples was determined using AOAC (2011) standardized methods. Dry matter (DM) was assessed by drying the samples at 105°C until a constant weight was achieved. CP was measured using the Kjeldahl nitrogen determination method. EE was analyzed by Soxhlet extraction with petroleum ether. The ash content was measured by incinerating the samples for 6 h at 550°C in a muffle furnace.

### 2.5. Economy of Production

Economic analysis was calculated according to [27, 28]. Feed cost (EGP/bird) was calculated based on FI and the cost of the experimental diets, including DDGS. Total cost included feed cost and other production costs. Net profit was calculated as the difference between total profit and total cost. Economic efficiency (%) was calculated as follows: (net profit/total cost) × 100, while relative economic efficiency (%) was calculated as follows: (economic efficiency of the treated group/economic efficiency of the control group) × 100.

### 2.6. Statistical Analysis

The results of all parameters (immune modulation, antioxidant and anti‐inflammatory effects, growth performance, carcass traits, blood hematology, serum parameters, meat quality, and economic efficiency) in the current study were tested for the statistical significance of DDGS levels by one‐way ANOVA, followed by Duncan’s multiple range tests, the SPSS 2023 software (SPSS, Inc., Chicago, IL, 2023) [29], with significance set at *p* < 0.05. The data for each variable are presented as means with pooled standard errors.

The experiment was analyzed according to the following statistical model:

(1) Yij=μ+Ti+eij,

where *Y*_ij_ is the observed value, *μ* is the overall mean, *T*_*i*_ is the fixed effect of DDGS, and *e*_ij_ is the random error.

## 3. Results

### 3.1. Growth Performance

The incorporation of DDGS in the diet improved live weight at 5‐week age (*p* < 0.05), and Groups 4 and 5 showed the highest values (2247 and 2240 g, respectively) relative to the control (1970 g) and other groups shown in Table 2. Moreover, weight gain, FCR, and production index were improved (*p* < 0.001) throughout the period with DDGS compared to the control group. Nonetheless, FI was markedly elevated (3261, 3293, 3297, and 3324 g/bird for G2, G3, G4, and G5, respectively; *p* < 0.001) in all DDGS groups relative to the control group (3214 g/bird). Nonetheless, a substantial drop in the FI of the G4 and G5 was observed in comparison to the other experimental groups on Day 35.

**TABLE 2:** Effects of dietary treatments on the growth performance of broiler chickens.

Variables	Groups (%DDGS)						
	1 (0)	2 (8)	3 (16)	4 (24)	5 (32)	SEM	*p* value
Body weight (g/bird)							
1 day	50.20	50.46	51.40	48.96	49.90	0.39	0.13
3 weeks	1138	1180	1188	1193	1193	10.28	0.068
5 weeks	1970^b^	2147^a^	2181^a^	2247^a^	2240^a^	19.25	< 0.001
Bodyweight gain (g/bird)							
1 day–3 weeks	1088	1130	1137	1144	1143	10.33	0.070
3–5 weeks	831^b^	966^a^	992^a^	1054^a^	1047^a^	13.03	< 0.001
Overall	1920^b^	2097^a^	2130^a^	2198^a^	2190^a^	20.75	< 0.001
Feed intake (g/bird)							
1 day–3 weeks	1105^d^	1169^c^	1172^c^	1212^b^	1240^a^	0.97	< 0.001
3–5 weeks	2251^a^	2092^c^	2121^b^	2085^d^	2083^d^	0.34	< 0.001
Overall	3214^d^	3261^c^	3293^b^	3297^b^	3324^a^	0.55	< 0.001
Feed conversion ratio (g feed/g gain)							
1 day–3 weeks	1.02^b^	1.03^b^	1.03^b^	1.06^ab^	1.09^a^	0.01	< 0.001
3–5 weeks	2.76^a^	2.20^b^	2.19^b^	2.02^b^	2.05^b^	0.04	< 0.001
Overall	1.69^a^	1.56^a^	1.56^b^	1.51^b^	1.53^b^	0.01	0.001

*Note:* Means with different superscripts in the same row differ significantly (*p* < 0.05).

### 3.2. Carcass Traits

As shown in Table 3, the results clarified that dietary DDGS did not affect the dressing % and the relative weight of heart, gizzard, liver, and spleen. On the other hand, the percentages of the bursa of Fabricius were improved (*p* < 0.001) in all DDGS‐supplemented groups relative to the control. Moreover, thymus percentages were improved (*p* < 0.001) in all DDGS‐supplemented groups relative to the control.

**TABLE 3:** Effects of dietary treatments on carcass traits and immune organs of broiler chickens.

Variables	Groups (%DDGS)						
	1 (0)	2 (8)	3 (16)	4 (24)	5 (32)	SEM	*p* value
Carcass traits, %							
Dressing	82.22	81.12	80.60	80.82	79.24	1.440	0.98
Heart	0.46	0.48	0.55	0.53	0.53	0.015	0.36
Gizzard	1.05	1.04	1.21	1.26	1.21	0.035	0.17
Liver	2.60	2.52	2.56	2.65	2.46	0.090	0.99
Spleen	0.10^b^	0.10^b^	0.15^a^	0.12^ab^	0.15^a^	0.019	0.003
Bursa	0.05^b^	0.06^b^	0.09^ab^	0.12^a^	0.14^a^	0.013	0.001
Thymus	0.45^b^	0.60^ab^	0.63^ab^	0.62^ab^	0.72^a^	0.069	0.005

*Note:* Means with different superscripts in the same row differ significantly (*p* < 0.05).

### 3.3. Hematological Parameters

Dietary inclusion of DDGS significantly affected RBC and WBC counts. The G3 exhibited the highest RBC count (2.78 × 10^6^/μL vs. 2.77 × 10^6^/μL in the control), whereas the G4 showed the highest WBC count (31.40 × 10^3^/μL vs. 30.93 × 10^3^/μL in the control) (Table 4). Significant differences were detected in Hb concentration between the treated groups (*p* < 0.001), with the group fed 16% DDGS showing the highest levels. Moreover, a significant difference was detected in HET, with G5 presenting increased levels. Moreover, there was a significant elevation in LYM and a drop in HET and the HET/LYM ratio with 16% DDGS supplementation in the diet (*p* < 0.05). Furthermore, groups fed 16% and 24% DDGS had higher MON counts, increasing by 17.2% and 13.8%, respectively, than those fed the control diet. There were no significant variations in ESO and BAS between the different dietary treatments (*p* < 0.05).

**TABLE 4:** Effects of dietary treatments on hematological indices of broiler chickens.

Variables	Groups (%DDGS)						
	1 (0)	2 (8)	3 (16)	4 (24)	5 (32)	SEM	*p* value
Hematological indices							
RBCs (× 10^6^/μL)	2.77^a^	2.54^b^	2.78^a^	2.76^a^	2.49^b^	0.020	0.03
WBCs (× 10^3^/μL)	30.93^a^	26.47^c^	30.67^a^	31.40^a^	26.87^c^	0.162	0.001
Hb (g/dL)	11.29^a^	11.37^a^	11.57^a^	11.35^a^	10.37^b^	0.055	0.001
PCV (%)	34.01^a^	27.50^c^	33.37^a^	32.28^b^	29.44^bc^	0.045	0.001
HET (× 10^3^/μL)	31.00^a^	32.50^a^	28.67^b^	32.00^a^	33.00^a^	0.221	0.01
LYM (× 10^3^/μL)	50.00b^c^	50.83^b^	55.33^a^	50.33^bc^	49.00^c^	0.200	0.001
HET/LYM ratio	0.62^b^	0.63^ab^	0.52^c^	0.64^ab^	0.67^a^	0.007	0.001
MON (× 10^3^/μL)	9.67^b^	10.67^ab^	11.33^a^	11.00^a^	10.67^ab^	0.138	0.009
ESO (× 10^3^/μL)	4.67^b^	5.33^a^	5.00^a^	5.33^a^	4.67^b^	0.143	0.03
BAS (× 10^3^/μL)	0.67	0.67	1.00	0.67	0.67	0.133	0.22

*Note:* RBCs, red blood cells; WBCs, white blood cells; Hb, hemoglobin; HET, heterophil; LYM, lymphocyte; MON, monocyte; ESO, eosinophil; BAS, basophil. Means with different superscripts in the same row differ significantly (*p* < 0.05).

Abbreviation: PCV, packed cell value.

### 3.4. Serum Metabolites

As shown in Table 5, dietary inclusion of DDGS significantly affected serum concentrations of TP and GLOB (*p* < 0.001), with birds fed the diet containing 8% DDGS exhibiting the highest levels. However, the albumin level revealed no significant variation. The lowest serum uric acid levels, representing a 15.7% decrease compared to the control group, were substantially (*p* < 0.05) recorded for broiler chicks fed diets containing 32% DDGS. The serum concentrations of triglycerides, cholesterol, and HDL‐C were significantly lowered (*p* < 0.001) in birds fed diets fortified with DDGS and Group G5, which was fortified with 32% DDGS, revealing decreased triglyceride and cholesterol levels. While G2, fortified with 8% DDGS, revealed a 15.3% decrease in HDL‐C levels compared to the control group. The highest concentrations of triiodothyronine (T3) and thyroxin (T4) were statistically observed (*p* < 0.001) in the serum of the broilers fed 32% and 24% DDGS, respectively.

**TABLE 5:** Effects of dietary treatments on serum metabolites of broiler chickens.

Serum metabolites	Groups (%DDGS)						
	1 (0)	2 (8)	3 (16)	4 (24)	5 (32)	SEM	*p* value
TP (g/dL)	3.54^ab^	4.25^a^	3.45^ab^	3.35^b^	3.10^b^	0.049	< 0.001
ALB (g/dL)	1.03	1.04	1.03	0.99	0.92	0.014	0.070
GLO (g/dL)	2.51^b^	3.22^a^	2.41^b^	2.39^b^	2.21^b^	0.079	< 0.001
A/G ratio	0.41^a^	0.32^b^	0.43^a^	0.42^a^	0.41^a^	0.017	0.02
Uric acid (mg/dL)	5.23^a^	5.51^a^	5.37^a^	5.01^ab^	4.41^b^	0.089	0.02
TRI (mg/dL)	113^ab^	120^a^	111^b^	103^bc^	92^c^	0.308	< 0.001
CHO (mg/dL)	100.11^a^	74.00^b^	60.07^c^	80.53^ab^	62.93^c^	0.110	< 0.001
HDL‐C (mg/dL)	50.60^a^	35.03^c^	41.10^b^	42.15^b^	35.87^c^	0.186	< 0.001
T3 (ng/mL)	4.54^b^	3.51^c^	4.85^ab^	4.97^ab^	5.32^a^	0.042	< 0.001
T4 (ng/mL)	21.29^c^	22.56^bc^	23.83^ab^	24.24^a^	23.68^ab^	0.213	< 0.001

*Note:* ALB, albumin; TR, triglycerides; CHO, cholesterol; T3, triiodothyronine, T4, thyroxin; GLO, globulin. Means with different superscripts in the same row differ significantly (*p* < 0.05).

Abbreviations: HDL‐C, high‐density lipoprotein cholesterol; TP, total protein.

### 3.5. Antioxidant and Immunological Status

The groups supplemented with DDGS have considerably higher levels (*p* < 0.001) of GPX and SOD and decreased MDA levels (*p* < 0.001) than the control group, as presented in Table 6, and G5, which was fortified with 32% DDGS, revealed a 66.8% increase in GPX levels, whereas G2, which was fortified with 8% DDGS, revealed a 44.2% increase in SOD levels and a 48.8% decrease in MDA levels in comparison to the control group. Inclusion of DDGS significantly impacts serum IFN‐γ, IL‐10, and C3 levels (*p* < 0.001). G3 revealed increased IFN‐γ, G4 revealed increased IL‐10 levels, whereas G5 revealed improved C3 levels. The serum IgA concentration was substantially higher in the G2 and G3 groups (*p* < 0.001). Serum IgG was substantially highest (*p* < 0.001) in birds fed DDGS at 8%. The serum IgM level was substantially increased (*p* < 0.05) due to DDGS inclusion at 8% and 32%.

**TABLE 6:** Effects of dietary treatments on antioxidant and immunological status of broiler chickens.

Serum metabolites	Groups (%DDGS)						
	1 (0)	2 (8)	3 (16)	4 (24)	5 (32)	SEM	*p* value
GPX (U/mL)	60.38^c^	100.33^a^	95.27^b^	96.27^b^	100.73^a^	0.228	< 0.001
SOD (U/mL)	22.19^c^	32.00^a^	30.00^b^	30.45^b^	22.26^c^	0.803	< 0.001
MDA (nmol/mL)	10.63^a^	5.44^c^	6.95^b^	6.53^bc^	8.13^ab^	0.117	< 0.001
IFN‐γ (pg/mL)	5.40^a^	4.61^b^	5.61^a^	3.60^c^	4.90^ab^	0.041	< 0.001
IL ‐10 (pg/mL)	1.65^c^	2.43^c^	3.14^b^	4.12^a^	3.86^ab^	0.017	< 0.001
C3 (g/L)	1.13^c^	1.30^b^	1.36^ab^	1.37^ab^	1.42^a^	0.008	< 0.001
IgA (mg/dL)	63.00^b^	65.00^a^	66.00^a^	61.71^bc^	59.16^c^	0.241	< 0.001
IgG (mg/dL)	871.33^a^	880.33^a^	830.67^b^	865.73^a^	779.38^c^	1.322	< 0.001
IgM (mg/dL)	23.41^b^	24.76^a^	23.77^ab^	21.52^c^	24.81^a^	0.181	< 0.001

*Note:* GPX, glutathione peroxidase; SOD, superoxide dismutase; MDA, malondialdehyde; IFN‐γ, interferon‐gamma; IL‐10, interleukin‐10; C3, Complement 3 proteins; IgA, immunoglobulin A; IgG, immunoglobulin G; IgM, immunoglobulin M. Means with different superscripts in the same row differ significantly (*p* < 0.05).

### 3.6. Chemical Composition of the Breast and Thigh Meat

In breast meat, significant differences were found in DM and ash content, while CP and EE levels remained statistically unaffected (Table 7). The content of DM differed significantly among the groups (*p* < 0.001), with the G5 exhibiting the highest value, representing a 5.6% increase compared to the control group. For ash content, G1 again recorded the lowest value, which was significantly different (*p* < 0.05) from all other groups. However, no significant variation was observed in CP or EE, indicating that the treatments did not influence protein or fat deposition in the breast meat (*p* < 0.05).

**TABLE 7:** Effects of dietary treatments on proximate composition of chicken breast and thigh meat.

Variables	Groups (%DDGS)						
	1 (0)	2 (8)	3 (16)	4 (24)	5 (32)	SEM	*p* value
Breast meat							
Dry matter, %	25.47^c^	26^bc^	26.53^ab^	25.73^c^	26.90^a^	0.068	< 0.001
Protein, %	85.40^a^	85.33^a^	84.78^b^	85.44^a^	84.83^b^	0.071	0.004
Fat, %	11.13^ab^	11.07^ab^	11.60^a^	10.89^b^	11.55^ab^	0.075	0.02
Ash, %	3.47^b^	3.61^a^	3.62^a^	3.67^a^	3.62^a^	0.015	< 0.001
Thigh meat							
Dry matter, %	23.40^c^	24.13^b^	24.03^b^	23.87^b^	24.90^a^	0.047	< 0.001
Protein, %	84.77^ab^	84.99^a^	84.12^ab^	84.25^ab^	83.64^b^	0.113	0.007
Fat, %	11.41^b^	11.76^ab^	12.27^ab^	12.13^ab^	12.76^a^	0.131	0.01
Ash, %	3.48^b^	3.59^ab^	3.60^a^	3.62^a^	3.60^a^	0.011	0.002

*Note:* Means with different superscripts in the same row differ significantly (*p* < 0.05).

In thigh meat, a statistically significant change (*p* < 0.001) was detected in DM content (Table 7). G5 recorded the highest DM value, representing a 6.4% increase compared to the control and significantly (*p* < 0.001) greater than all other DDGS groups. Conversely, G1 had the lowest DM content. The results suggest that the treatments had a substantial effect on the moisture‐related composition of the thigh meat. No statistically significant changes were detected among the groups (*p* < 0.05) for CP, EE, and ash content. However, ash content tended toward significance (*p* < 0.05), with G1 having a lower value than the others.

### 3.7. Economic Efficiency

Table 8 provides a comparative economic evaluation of the experimental diets, detailing feed costs and returns. Compared to the control group, birds fed 32% DDGS showed an increase in net revenue, along with improvements in economic efficiency and relative economic efficiency, followed by those fed 24%, 16%, and 8% DDGS, respectively.

**TABLE 8:** Economic analysis of broiler chickens as influenced by dietary treatments.

Variables	Groups (%DDGS)				
	1 (0)	2 (8)	3 (16)	4 (24)	5 (32)
Total feed cost, EGP	80.25	78.63	76.53	73.59	71.31
Total production cost, EGP	110.25	108.63	106.53	103.59	101.31
Total profit, EGP	138.92	150.68	154.72	157.35	158.70
Net profit, EGP	28.67	42.05	48.20	53.77	57.39
Economic efficiency	26.00	38.71	45.24	51.90	56.64
Relative economic efficiency	100.00	148.87	174.00	199.63	217.86

*Note:* EGP, Egyptian pounds.

## 4. Discussion

The improvement in all measured growth performance parameters in birds fed on diets supplemented with DDGS matches the outcomes of Min et al. [30], who recorded that broilers supplemented with 15% DDGS had higher average daily gains and lower FCR. Damasceno et al. [3] stated that broiler growth performance was unaffected by raising the DDGS content in their diets to 16%. Ravindran and Singh [31] reported that as DDGS inclusion levels increased, weight gain tended to improve during the 35‐day trial period. However, the authors in [32] found that compared to diets without DDGS supplementation, broiler diets fortified with 12, 18, or 24% DDGS had a detrimental impact on BWG and FCR. Valentim [33] noted that the productive performance of broilers did not differ significantly by dietary inclusion of DDGS. Jin et al. [34] reported a significant reduction in the BWG and FI of broilers by adding DDGS to the diet at 10, 15, or 20%. The DDGS’s beneficial effect on BWG and FCR may be explained by its abundance of calories, protein, and amino acids.

Regarding the relative weight of the internal organs, the present trial explained that dietary treatment with DDGS improved the relative weights of the bursa of Fabricius and thymus, which are crucial organs for the immune system. These findings suggest that DDGS may positively impact the immune function of broilers, potentially enhancing their resistance to diseases. Our conclusions are consistent with those of Youssef et al. [35], who found that the weight of organs such as the heart, liver, and gizzard was not significantly affected by adding 10 or 15% DDGS as a protein source to broiler diets. Similarly, the authors in [32] noted that the percentages of broiler chickens’ dressing, liver, and heart were unaffected by the addition of DDGS to their diet. Shim and Kim et al. [36, 37] confirmed our findings, noting no significant changes in the carcass yield of broilers due to DDGS supplementation.

However, the authors in [38] noted that carcass yields were significantly impacted by the dietary supplementation of DDGS up to 20% in the broiler’s diet. Borah [39] demonstrated that different levels of DDGS inclusion in the diets did not impact the weights of the gizzard, heart, and liver. Elbaz [40] reported a significant decline in the percentage of the bursa of Fabricius with dietary inclusion of DDGS up to 30%. The observed discrepancies among studies may be attributed to variations in the DDGS source, processing methods, inclusion level, and nutrient composition, as well as differences in diet formulation, dietary energy balance, broiler genotype, bird age, feeding duration, and experimental conditions.

Our findings regarding the outcome of supplementing DDGS to broilers’ diet on their hematological parameters were in agreement with Hristakieva et al. [41], who found that broilers fed 15% DDGS had the highest RBC count values (*p* < 0.05) when compared to other treatments. Furthermore, our findings differ from those of Ghaly [42], who reported that at 5%, 10%, and 15% levels of DDGS in the broiler diet, no significant differences were observed in WBC and RBC counts. Ugale [43] demonstrated significant differences in PCV due to DDGS supplementation. Roper et al. [44] stated that the addition of DDGS to the diet did not significantly impact broiler MON counts. However, Gupta et al. [45] reported that dietary supplementation of DDGS at levels exceeding 5% significantly (*p* < 0.05) elevated the levels of PCV and Hb in broiler chickens. The decrease in PCV% and Hb concentration in broilers fed DDGS at 32% may be attributed to the high fiber content, which negatively affects nutrient digestibility and potentially introduces antinutritional factors that decrease nutrient absorption [32]. The noted increase in LYM and the decrease in HET counts, as well as the HET/LYM ratio, with 16% DDGS supplementation, imply that this level of DDGS inclusion may have a beneficial impact on the immune system of broilers, potentially enhancing immune response and reducing stress or inflammation [30].

The present results show that adding DDGS to the broiler chickens’ diet had varying impacts on their serum biochemical indices. Since globulins comprise immunoglobulins and other immune proteins, the significant increase in globulin levels in birds given 8% DDGS indicated an enhancement in immune‐related protein synthesis. In contrast, no significant changes were detected in serum total protein, albumin, or the A/G ratio. This is consistent with [18], who demonstrated that moderate DDGS inclusion may enhance humoral immunity due to yeast‐derived β‐glucans and mannans present in DDGS. Similarly, the authors in [46] reported that total protein concentrations were unaffected by the amount of DDGS included in laying hens’ diets. Kaninde [47] mentioned no significant change in blood total protein or albumin levels when broiler diets were supplemented with DDGS. Serum uric acid and TRI levels were significantly lower in broilers fed 32% DDGS. The reduction in uric acid may reflect improved nitrogen utilization or reduced protein catabolism. Lower triglyceride levels may be attributed to fiber content and unsaturated fatty acids in DDGS, which modulate lipid metabolism and fat absorption [48].

In addition, birds fed DDGS showed significantly reduced serum CHO and HDL levels, consistent with reports that DDGS reduces blood lipids via its polyunsaturated fatty acid content and potential modulation of hepatic lipid synthesis [49]. Dinani [50] demonstrated that the serum cholesterol levels were significantly reduced when broiler chickens were fed 15% DDGS. Elbaz et al. [40] noted that plasma total cholesterol concentrations were significantly reduced by dietary supplementation of DDGS into broilers’ diet. Moreover, the highest concentrations of G3 and G4 were recorded in birds fed 32% and 24% DDGS, respectively. This indicates stimulation of thyroid activity, possibly due to increased metabolic demand or stress at higher DDGS levels. Zhang et al. [51] stated that the blood level of G3 in ducks fed diets fortified with DDGS at 5%, 8%, and 11% DDGS was markedly less than that in the control group, although blood T4 levels were considerably elevated. However, the authors in [42] stated that the dietary inclusion of DDGS at 5%, 10%, and 15% into broiler diets had no impact on their serum T3 and T4 levels.

The notable elevation of GPX and SOD levels in broilers fed DDGS indicates an enhanced antioxidant defense response. DDGS contains bioactive components, including natural antioxidants such as tocopherols, selenium, and phenolic compounds, which may upregulate endogenous antioxidant enzymes [52]. The improved antioxidant status helps neutralize reactive oxygen species, reducing oxidative stress and supporting overall metabolic health. The significant decrease in blood MDA and IFN‐γ levels in broilers fed with DDGS suggests a decrease in oxidative stress and inflammatory response [18]. Min et al. [30] reported that the inclusion of DDGS decreased SOD, whereas it increased MDA in broiler serum and had no effect on GSH‐Px levels. Ruan et al. [53] noted that the plasma concentration of GSH in broilers was unaffected by corn DDGS at concentrations of 10%, 20%, or 30%.

The positive effect of DDGS on the broilers’ serum of IL‐10 and C3 suggests a dual enhancement of anti‐inflammatory and innate immune responses [54]. The significant improvement in serum IgA in the G2 and G3 groups, and the decrease in IgG in birds fed 32% DDGS‐fortified diets, may suggest immune suppression or altered B‐cell function at high inclusion rates. This could be linked to mycotoxin exposure or nutritional imbalances affecting protein metabolism and antibody synthesis [55]. On the other hand, IgM levels were significantly elevated in birds fed 8% and 32% DDGS, indicating activation of primary immune responses. The increase at 8% likely reflects an optimal immunostimulant, while the rise at 32% could be a compensatory response to dietary stress. Elbaz et al. [40] showed that increasing the quantity of DDGS in broiler diets negatively impacted IgM levels, although the inclusion levels of DDGS did not significantly modify IgG concentrations. In addition, broilers fed 5% and 10% DDGS had elevated (*p* < 0.05) concentrations of IgM.

The notable differences (*p* < 0.05) in DM content between the trial groups indicate that dietary DDGS influenced the chemical composition of breast meat. The elevated DM levels observed in broilers receiving moderate DDGS supplementation may reflect changes in meat composition. This finding agrees with Jin et al. [34], who reported that fiber‐rich byproducts such as DDGS can influence nutrient utilization and meat composition. Considering the relatively low starch and high protein and fiber contents of DDGS [56], these nutritional characteristics may have contributed to the observed changes in the DM content. Previous studies have suggested that DDGS may influence gut fermentation dynamics and osmotic balance [31, 57]. However, these parameters were not directly evaluated in the present study; therefore, their contribution to the observed changes remains speculative and warrants further investigation.

The CP content across the groups did not vary significantly, indicating that the diets were well balanced in terms of protein equivalence. This outcome mirrors findings by Ajao et al. [58], who noted no adverse impact on carcass CP levels when soybean meal was partially replaced with DDGS or canola meal in iso‐nitrogenous formulations. Protein deposition depends heavily on digestible amino acids and the overall energy supply; thus, the uniformity in CP levels across treatments underscores the effectiveness of dietary formulation and adequate amino acid profiles [59]. Furthermore, the residual yeast and fermentation byproducts in DDGS may have supported nutrient absorption and gut health through prebiotic actions, fostering protein synthesis even when conventional protein inputs were reduced [60].

While not statistically significant, numerical trends revealed greater EE levels in thigh muscles than in breast tissue, a pattern consistent with the metabolic and anatomical characteristics of these muscles. Thigh muscles, being more oxidative, tend to accumulate more lipids compared to the glycolytic muscles in the breast [51]. The residual fat and unsaturated lipids in DDGS [61] could influence fat deposition, though the actual effect may vary depending on how efficiently the birds utilized dietary energy. Fiber‐induced reduction in fat digestibility could also play a role in tempering lipid storage [34].

Significant variations in ash content across treatments indicate differences in mineral retention, likely influenced by fiber levels. Reduced ash concentrations at higher DDGS inclusions may stem from dietary fiber interfering with the absorption of key minerals, including calcium and phosphorus. According to Świątkiewicz and Koreleski [62], nonstarch polysaccharides (NSPs) in DDGS can bind to minerals, impairing their bioavailability. Moreover, the increased gut transit speed often caused by high fiber intake could further reduce mineral absorption efficiency by limiting exposure time in the intestine. This finding underscores the importance of supplementing with proper phytase or chelated minerals when high‐DDGS diets are used [57]. The contrasting responses between breast and thigh meat underscore muscle‐specific physiological differences. While breast meat is denser in protein and has lower vascularization, thigh meat is metabolically more active and has greater fat storage capacity. The consistent CP and EE values across all treatments suggest that energy and protein requirements for tissue development were adequately met, despite changes in protein source [51].

Improving final LBW and FCR with DDGS and decreasing the feed cost of the DDGS groups were the reasons for the higher recorded net profit and relative economic efficiency. As the concentration of DDGS was elevated in the broilers’ diet, lower feed costs per kg of feed were obtained. The same findings were reported by Jin et al. [34] when 15% and 30% DDGS were included in the broiler feed with and without enzyme treatment, and Tang et al. [63] stated that the cost per ton of feed reduced as the level of DDGS addition elevated. Furthermore, incorporating DDGS into the diet can substantially enhance economic advantages.

## 5. Conclusion

Under the conditions of the current study, the incorporation of DDGS in broiler diets up to 32% had no adverse impact on growth performance while improving immune response, antioxidant status, and meat quality. Moreover, DDGS significantly lowered feed costs and enhanced net profit, establishing it as a viable and cost‐effective alternative protein source for broiler nutrition. However, further research under different environmental conditions, production systems, and inclusion levels is needed to confirm the long‐term impacts and broader applicability of DDGS in the poultry industry.

## Author Contributions

All authors contributed equally to this manuscript.

## Funding

This work was supported by the Deanship of Scientific Research, Vice Presidency for Graduate Studies and Scientific Research, King Faisal University, Saudi Arabia [Grant no. KFU263889].

## Disclosure

All authors have read and agreed to the published version of the manuscript.

## Ethics Statement

The ethical statement adhered to the protocols established by the Ethical Committee of the Egyptian Research for the Use and Care of Laboratory Animals, as mandated by New Valley University (NVREC/02/3/9/2025/26). The study was carried out in compliance with the ARRIVE guidelines.

## Conflicts of Interest

The authors declare no conflicts of interest.

## Data Availability

The original contributions presented are included in the article. Further inquiries can be directed to the corresponding authors.
